# High-dimensional immune profiling of peripheral blood identifies immune correlates of anti-PD-1/PD-L1 resistance in oncogenic driver mutation-positive NSCLC

**DOI:** 10.3389/fimmu.2026.1829816

**Published:** 2026-05-29

**Authors:** Kyoo Hyun Kim, Mina Han, Geon Woo Park, Min Hee Hong, Gamin Kim, Brian Hyohyoung Lee, Wonrak Son, Jaehyung Kim, Seokhyeong Go, Jeongah Kim, Geoffrey Kelly, Kai Nie, Seunghee Kim-Schulze, Miriam Merad, Sangwoo Kim, Chang Gon Kim, Hyun Je Kim, Hye Ryun Kim

**Affiliations:** 1 Division of Medical Oncology, Department of Internal Medicine, Yonsei Cancer Center, Severance Hospital, Yonsei University College of Medicine, Seoul, Republic of Korea; 2 Division of Medical Oncology, Department of Internal Medicine, Yonsei University College of Medicine, Seoul, Republic of Korea; 3 Department of Biomedical Science, Seoul National University Graduate School, Seoul, Republic of Korea; 4 Department of Microbiology and Immunology, Seoul National University College of Medicine, Seoul, Republic of Korea; 5 Human Immune Monitoring Center, Icahn School of Medicine at Mount Sinai, New York, NY, United States; 6 Marc and Jennifer Lipschultz Precision Immunology Institute, Icahn School of Medicine at Mount Sinai, New York, NY, United States; 7 Department of Immunology and Immunotherapy, Icahn School of Medicine at Mount Sinai, New York, NY, United States; 8 The Tisch Cancer Institute, Icahn School of Medicine at Mount Sinai, New York, NY, United States; 9 Department of Biomedical Systems Informatics, Brain Korea 21 PLUS Project for Medical Science, Yonsei University College of Medicine, Seoul, Republic of Korea; 10 Cancer Research Institute, Seoul National University College of Medicine, Seoul, Republic of Korea; 11 Genomic Medicine Institute, Medical Research Center, Seoul National University College of Medicine, Seoul, Republic of Korea; 12 Interdisciplinary Program in Artificial Intelligence (IPAI), Seoul National University, Seoul, Republic of Korea

**Keywords:** anti PD-1/PD-L1 monoclonal antibody, cyTOF, cytometry by time-of-flight, Immune profiling, non-small cell lung cancer (NSCLC), PBMC (peripheral blood mononuclear cells)

## Abstract

**Background:**

Non-small cell lung cancer (NSCLC) patients with oncogenic driver mutations such as *EGFR*, *ALK* or *ROS1* (mutant-type [MT]) exhibit poor responses to PD-1/PD-L1 immune checkpoint inhibitors (ICIs) compared to wild-type (WT) patients. The mechanisms underlying this limited response to ICIs in MT patients remain unclear. This study aimed to identify key immune biomarkers and elucidate immune cell dynamics in peripheral blood contributing to ICI resistance in MT-NSCLC.

**Methods:**

A total of 262 NSCLC patients who received PD-1/PD-L1 inhibitor monotherapy between February 2018 and July 2024 were included. Of these, 43 patients were assigned to the discovery cohort, where immune profiling was performed on peripheral blood mononuclear cells using Cytometry by Time-of-Flight (CyTOF) at baseline and cycle 2, day 1 (C2D1). The findings were validated in an independent cohort (n=57) using flow cytometry.

**Results:**

Baseline CXCR3^+^ CD127^+^ effector CD8^+^ T cells were significantly elevated in WT compared to MT patients (*P* = 0.0120) and were a robust predictor of favorable response (AUC = 0.745). Patients with CXCR3^+^ CD127^+^ CD8^+^ T cell frequencies above 3.54% exhibited superior progression-free survival (PFS, *P* = 0.0005) and overall survival (OS, *P* = 0.0012). In contrast, MT patients demonstrated a distinct reduction in CD27^+^ PD-1⁻ effector memory CD4^+^ T cells during treatment, correlating with poor outcomes (AUC = 0.716). This reduction was associated with diminished conventional dendritic cell (cDC) abundance, suggesting impaired T-cell differentiation and function in MT patients. These findings were consistently validated using flow cytometry in an independent cohort.

**Conclusion:**

Distinct immune cell profiles highlight elevated baseline CXCR3^+^ CD127^+^ effector CD8^+^ T cells predicting favorable outcomes and impaired cDC-CD4^+^ T cell dynamics as critical contributors to anti-PD-1/PD-L1 resistance in MT-NSCLC.

## Background

Lung cancer remains the leading cause of cancer-related mortality worldwide, accounting for approximately 1.8 million deaths annually, despite recent declines in mortality rates (1, 2). In East Asia, up to 50% of non-small cell lung cancer (NSCLC) patients harbor epidermal growth factor receptor (*EGFR*) mutations (3), 5-7% have anaplastic lymphoma kinase (*ALK*) rearrangements (4), and 1% have *ROS1* proto-oncogene (*ROS1*) rearrangements (5). Patients of these mutant-types (MT) derive substantial survival benefits and experience lower toxicity from tyrosine kinase inhibitors (TKIs) compared to chemotherapy (6–8). However, acquired resistance to TKIs remains a major clinical challenge, underscoring the need for alternative therapeutic strategies.

Beyond targeted therapies, immune checkpoint inhibitors (ICIs) targeting programmed death-1 (PD-1) and programmed death ligand-1 (PD-L1) have significantly improved survival and quality of life for patients with advanced NSCLC (9–13). Currently, tumor PD-L1 expression, assessed through immunohistochemistry (IHC), serves as the primary biomarker for ICI selection (14). However, PD-L1 expression alone does not always correlate with clinical benefit and may not reliably predict ICI response in tumors harboring driver mutations such as *EGFR*, *ALK*, or *ROS1*.

Although anti-PD-1/PD-L1 agents, including nivolumab, pembrolizumab, and atezolizumab, have been FDA-approved for use in both first- or later-line settings for advanced NSCLCs, they are not recommended as frontline therapies for MT-NSCLC due to their limited survival benefit compared to wild-type (WT) NSCLC (15, 16). To address this limitation, alternative predictive biomarkers — such as tumor mutation burden (TMB), pre-existing T-cell infiltration, and transcriptomic signatures — have been proposed to enhance patient stratification for anti PD-1/PD-L1 therapy (14). Nonetheless, the identification of robust biomarkers that specifically underlie the poor prognosis and limited ICI response in MT-NSCLC patients remains an unmet clinical need.

Given the dynamic tumor immune microenvironment, there is a growing interest in identifying blood-based dynamic biomarkers capable of capturing immune system changes during treatment. In this context, peripheral blood mononuclear cells (PBMCs) provide a minimally invasive and clinically feasible tool for serial immune monitoring, allowing comprehensive assessment of immune dynamics throughout the course of therapy (17).

This study aims to identify predictive biomarkers and investigate immune cell dynamics in peripheral blood to assess responses to anti-PD-1/PD-L1 therapy in patients with WT- and MT-NSCLC. We examined immune cells in PBMCs from NSCLC patients at baseline and on Cycle 2 Day 1 (C2D1) of anti-PD-1/PD-L1 monotherapy using high-dimensional mass cytometry (CyTOF), with validation by flow cytometry in an independent cohort.

## Methods

### Study population and clinical data collection

A total of 262 patients with recurrent and/or metastatic NSCLC, who had received at least one dose of PD-1/PD-L1 inhibitor monotherapy (nivolumab, pembrolizumab, or atezolizumab) at Yonsei Cancer Center between February 2018 and July 2024, were identified from institutional biomarker trial databases. Clinical data, including patient characteristics, oncogenic driver mutation status, treatment response to immunotherapy, and PD-L1 expression level, were collected and systemically analyzed. Treatment response was assessed using the Response Evaluation Criteria in Solid Tumors (RECIST) version 1.1 and categorized as complete response (CR), partial response (PR), stable disease (SD), or progressive disease (PD). Durable clinical benefit (DCB) was defined as CR, PR, or SD sustained for a minimum duration of six months.

From this overall cohort, 43 were assigned to the discovery cohort and 57 to the validation cohort. These subsets were selected based on the availability of high-quality peripheral blood mononuclear cell (PBMC) samples suitable for high-dimensional immune profiling. Patients in the discovery cohort (nivolumab, n=12; atezolizumab, n=31) were enrolled in prospective biomarker clinical trials aimed at identifying predictive immune biomarkers in peripheral blood (NCT03486119 for nivolumab; NCT04312308 for atezolizumab).

Peripheral blood samples were obtained at baseline and after one cycle of an anti-PD-1 (nivolumab) or anti-PD-L1 (atezolizumab) therapy. In the discovery cohort, PBMCs were analyzed using Cytometry by Time-of-Flight (CyTOF), while flow cytometry was used for PBMC analysis in the independent validation cohort.

This study was approved by the Institutional Review Board (IRB) of Yonsei College of Medicine (IRB numbers: 4-2016-0678, 4-2017-0788, 4-2019-0948), and all patients provided written informed consent for clinical data and blood sample collection.

### PD-L1 immunohistochemistry

PD-L1 expression data were collected from clinical pathology records of patients in the study cohort. Archived formalin-fixed, paraffin-embedded (FFPE) surgical specimens or biopsies had previously been evaluated by immunohistochemistry (IHC) as part of routine diagnostic work-up using one of the following clinically approved assays: PD-L1 SP263 (Ventana Medical Systems, USA), Dako PD-L1 IHC 22C3 pharmDx (Agilent, USA), or VENTANA PD-L1 SP142 (Ventana Medical Systems, USA) assays (18). PD-L1 positivity was defined as tumor cell (TC) ≥ 1% for SP142 or tumor proportion score (TPS) ≥ 1% for 22C3 and SP263.

### PBMC isolation

PBMC suspensions were prospectively prepared from peripheral blood collected in EDTA tubes (Cat# 367525, Becton Dickinson Vacutainer) at predefined time points. Mononuclear cells were isolated using a density gradient isolation technique. Following isolation, the sample was viably cryopreserved in a mixture of plain RPMI-1640 (Cat# 10-040-CV, Corning), fetal bovine serum (FBS, Cat#16000-044, Gibco), and dimethyl sulfoxide (DMSO, Cat#D2650, Sigma). Cells were slowly frozen to maintain cell integrity and stored in liquid nitrogen.

### Sample preparation for high dimensional profiling of circulating immune cells

For CyTOF analysis, PBMCs were thawed as previously described (19). The cells were washed by centrifugation and incubated in a cell culture medium containing 1 μmol/L Cell-ID Rh103 nucleic acid intercalator (Cat# 201103A, Fluidigm Corporation) at 37 °C for 20 minutes to label the dead cells. Following incubation, the samples were washed and blocked with an Fc receptor–blocking solution (Cat #422302; BioLegend) to prevent non-specific antibody binding. Each sample from the same patient was subsequently barcoded using β2M antibody conjugated to a unique metal isotope. Barcoded samples were washed twice and pooled per patient to minimize staining variability between baseline and C2D1 time points for a given patient. Pooled samples were stained in a Cell Staining Buffer (CSB) containing a premade cocktail of cell surface antibodies at room temperature for 30 minutes. All antibodies were purchased commercially from Fluidigm or conjugated in-house using Fluidigm X8 polymer conjugation kits (**Supplementary Table 1**).

The pooled patient samples were then washed, fixed, and further barcoded using Fluidigm’s Cell-ID 20-Plex Pd barcoding kit (Fluidigm, Cat# 201060). To reduce variability in intracellular staining and instrument acquisition, the samples were washed twice and pooled. Next, the single pooled sample was fixed and permeabilized using the eBioscience FoxP3/transcription factor staining buffer kit (eBio, Cat# 00–5523–00), blocked with heparin (100 U/mL) to prevent non-specific binding to eosinophils (20), and stained with a premade cocktail of intracellular antibodies. Finally, the samples were incubated with freshly diluted 2.4% paraformaldehyde in PBS containing 0.02% saponin and 0.125 nmol/L Cell-ID Intercalator-Ir (Fluidigm, Cat# 201192A) at room temperature for 30 minutes for cell identification. The sample was then washed and stored in CSB at 4 °C until acquisition within 48 hours.

Immediately before the acquisition, the pooled sample was washed once with CSB and once with Cell Acquisition Solution (CAS) (Fluidigm, Cat# 201240) and resuspended in CAS at a concentration of 1 million cells/mL and a 1:20 dilution of EQ four-element normalization beads (Cat# 201078, Standard BioTools Inc.). Samples were acquired on a Fluidigm Helios mass cytometer using a wide-bore injector configuration at an acquisition rate of <400 cells/s (21). The resulting FCS files were normalized and concatenated using Fluidigm’s CyTOF software v7.0 and de-multiplexed using the Icahn School of Medicine at Mount Sinai Human Immune Monitoring Center’s in-house debarcoding pipeline (22).

### High-dimensional profiling of circulating immune cells by CyTOF

CyTOF analysis was performed on the entire sample cohort using Cytobank and Python. Using Cytobank, doublets were manually excluded, and live immune cells were identified based on CD45 and Ir-193 DNA expression. Subsequently, flow cytometry standard (FCS) files were exported for downstream analysis in Python. Raw expression data were normalized using arcsinh transformation (y=archsinh(x/5)). To integrate data across all samples, each sample was randomly down-sampled to 5,000 cells. Clustering and visualization were conducted using the Leiden algorithm and Uniform Manifold Approximation and Projection (UMAP), as implemented in the Scanpy Python package. Major immune populations were annotated based on canonical marker expression. Non-immune cells, identified by the absence of CD45 expression, were excluded. To identify specific subsets within each major immune population, clusters were further sub-clustered in the same approach. Differential expressions of features between groups were evaluated using the Wilcoxon rank-sum test, with Bonferroni correction applied for multiple comparisons.

### Multi-color flow cytometry

Cells were washed with cold Dulbecco’s Phosphate Buffered Saline with 2% Fetal Bovine Serum (STEMCELL, Cat# 07905) and stained with antibodies against cell-surface molecules for 20 min at 4 °C. The LLIVE/DEAD™ Fixable Near-IR dead cell stain kit (Invitrogen) was used to identify live cells. For intracellular staining, cells were fixed and permeabilized with the Foxp3 fixation/permeabilization solution (ThermoFisher Scientific) for 20 min at 4 °C, and stained with antibodies against intracellular molecules for 20 min at 4 °C. The stained cells were analyzed using FACS CytoFLEX (Beckman Coulter, Brea, California, USA) and the data were analyzed using FlowJo software V.10 (Treestar, San Carlos, California, USA). The antibodies used are listed in **Supplementary Table 2**. To normalize all samples, each sample was randomly down-sampled to 50,000 CD45^+^ lymphocytes (**Supplementary Figure 1**). Expression of CXCR3^+^ CD127^+^ effector CD8^+^ T cells and CD27^+^ PD-1^-^ effector memory CD4^+^ T cells from PBMCs was used to gate for CD45^+^ lymphocytes.

### Statistical analysis

Descriptive statistics summarized patient demographics, clinical characteristics, and outcomes. Categorical and continuous variables were compared using the chi-square test and unpaired t-tests, respectively. Progression-free survival (PFS) denoted the time from treatment initiation to disease progression or death, while overall survival (OS) measured the time from treatment initiation to death from any cause. Kaplan-Meier method was used for survival analysis, and survival curves were compared between groups using the log-rank test. Hazard ratios (HRs) and 95% confidence intervals (CIs) were estimated using the Cox proportional hazards model. Univariable and multivariable Cox regression analyses were performed to evaluate the independent prognostic significance of mutation status after adjusting for potential confounders.

For the CyTOF and flow cytometry statistical analyses, comparisons of cell frequencies between WT and MT groups at the same time point(baseline or C2D1) were performed using the two-sided Mann-Whitney U test. Longitudinal comparisons of paired samples from the same patients between baseline and C2D1 were performed using the Wilcoxon signed-rank test. CyTOF results were adjusted for multiple comparisons using the Benjamini–Hochberg method. All tests were two-tailed, and statistical significance was set at *P <* 0.05.

## Results

### Baseline demographic and clinical characteristics

A total of 262 patients with recurrent and/or metastatic NSCLC who received anti-PD-1/PD-L1 monotherapy between February 2018 and July 2024 and were included for clinical data analysis. From this cohort, 100 patients were selected for immune profiling analysis (discovery cohort, n=43; validation cohort, n=57). As all samples were prospectively collected within pre-registered biomarker clinical trials, the selection process was primarily governed by PBMC sample availability and quality, with inclusion of both mutation status groups (MT and WT) and both clinical responders and non-responders. Patients were stratified into WT or MT groups based on oncogenic driver mutations, including *EGFR*, *ALK*, or *ROS1* rearrangements.

**Figure 1:**
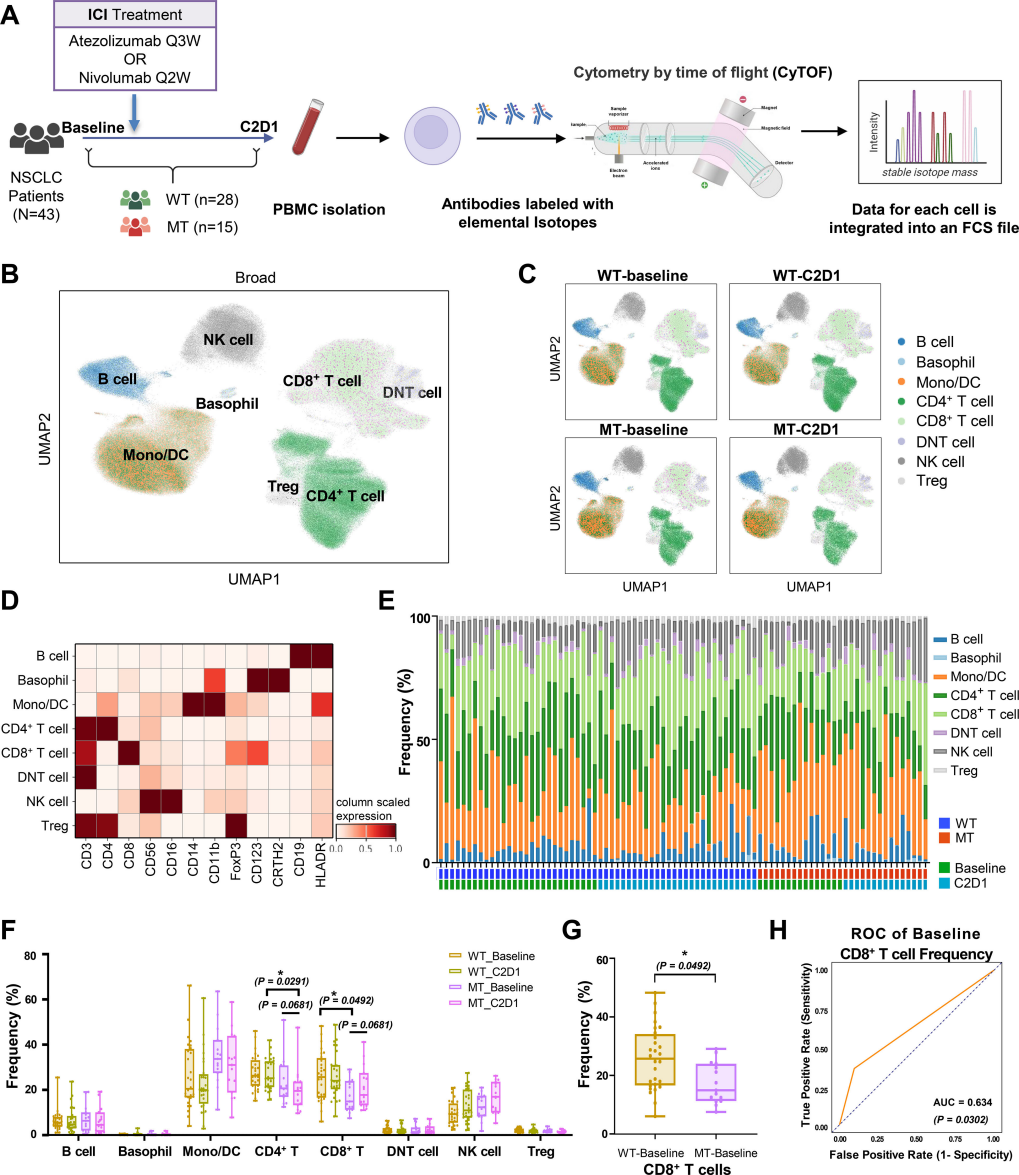
Clinical outcomes of anti-PD-1/PD-L1 monotherapy in wild-type and mutant NSCLC. **(A–D)** Kaplan-Meier curves for progression-free survival (PFS) in the retrospective **(A)** and discovery **(C)** cohorts, and overall survival (OS) in the retrospective **(B)** and discovery **(D)** cohorts, stratified by mutation status (WT, wild-type; MT, mutant). Hazard ratios (HR) and 95% confidence intervals (CI) were calculated using univariate Cox proportional hazards regression. **(E)** Proportion of patients achieving durable clinical benefit (DCB) versus no durable benefit (NDB) in the retrospective cohort. (*P=0.0006*) **(F)** Distribution of PD-L1 expression levels in retrospective cohort. (ns, not significant) **(G)** Receiver operating characteristic (ROC) curve evaluating the predictive performance of PD-L1 expression for treatment response in the overall, retrospective cohort. (AUC = 0.58; N = 262). ***P < 0.001; ns, not significant.

Baseline clinical characteristics (**Table 1**) showed that the WT group comprised more males (82.3% vs. 47.1%) and older patients aged ≥65 years (55.7% vs. 31.4%), while the MT group included more females, younger individuals, never-smokers (55.7%), and a higher prevalence of adenocarcinoma (LUAD: 90% [MT] vs. 57.3% [WT]). Clinical outcomes and PD-L1 characteristics are illustrated in **Figure 1**. PFS and OS were significantly shorter in the MT group (*P<0.001*; **Figure 1A**, *P =0.0166;* **Figure 1B**), and multivariable Cox regression confirmed MT status as an independent predictor of shorter PFS (HR 1.80, 95% CI 1.24–2.61; *P=0.002*), with a consistent but non-significant trend for OS (HR 1.44, 95% CI 0.97–2.13; *P=0.069*; **Supplementary Table 3**). The WT group also demonstrated higher objective response rates and a greater likelihood of achieving DCB compared to the MT group (*P = 0.0006;* **Figure 1E**).

**Table 1:** Baseline and treatment characteristics of NSCLC patients treated with ICI monotherapy.

Characteristics	Total patients N = 262						Discovery cohort (CyTOF) N = 43				Validation cohort (FACS) N = 57			
	Total (N = 262)		WT (N = 192)		MT (N = 70)		WT (N = 28)		MT (N = 15)		WT (N = 39)		MT (N = 18)	
	N	%	N	%	N	%	N	%	N	%	N	%	N	%
Gender														
Male	191	78.6	158	82.3	33	47.1	24	85.7	6	40.0	32	82.1	7	38.9
Female	71	29.2	34	17.7	37	52.9	4	14.3	9	60.0	7	17.9	11	61.1
Age (years)														
<65	133	54.7	85	44.3	48	68.6	11	39.3	10	66.7	14	35.9	13	72.2
≥65	129	53.1	107	55.7	22	31.4	17	60.7	5	33.3	25	64.1	5	27.8
Smoking														
Current	91	37.4	80	41.7	11	15.7	8	28.6	0	0.0	20	51.3	4	22.2
Former	93	38.3	73	38.0	20	28.6	18	64.3	5	33.3	11	28.2	4	22.2
Never	75	30.9	36	18.8	39	55.7	2	7.1	10	66.7	8	20.5	10	55.6
Unknown	3	1.2	3	1.6	–	–	–	–	–	–	–	–	–	–
Histological type														
LUAD	173	71.2	110	57.3	63	90.0	10	35.7	15	100.0	18	46.2	18	100.0
LUSC	76	31.3	73	38.0	3	4.3	15	53.6	0	0.0	18	46.2	–	–
Others	13	5.3	9	4.7	4	5.7	3	10.7	0	0.0	3	7.7	–	–
Clinical benefit														
DCB	92	37.9	79	41.1	13	18.6	20	71.4	2	13.3	14	35.9	3	16.7
NDB	168	69.1	111	57.8	57	81.4	8	28.6	13	86.7	25	64.1	15	83.3
Unknown	2	0.8	2	1.0	–	–	–	–	–	–	–	–	–	–
Best-Response														
CR	1	0.4	1	0.5	–	–	1	3.6	0	0.0	0	0.0	0	0.0
PR	49	20.2	40	20.8	9	12.9	8	28.6	1	6.7	4	10.3	2	11.1
SD	92	37.9	76	39.6	16	22.9	11	39.3	4	26.7	15	38.5	2	11.1
PD	118	48.6	73	38.0	45	64.3	8	28.6	10	66.7	20	51.3	14	77.8
Unknown	2	0.8	2	1.0	–	–	–	–	–	–	–	–	–	–
Stage at diagnosis														
I	27	11.1	18	9.4	9	12.9	3	10.7	1	6.7	5	12.8	3	16.7
II	15	6.2	12	6.3	3	4.3	2	7.1	1	6.7	3	7.7	0	0.0
III	76	31.3	65	33.9	11	15.7	8	28.6	3	20.0	13	33.3	2	11.1
IV	144	59.3	97	50.5	47	67.1	15	53.6	10	66.7	18	46.2	13	72.2
Drug														
Atezolizumab	119	49.0	81	42.2	38	54.3	20	71.4	11	73.3	19	48.7	11	61.1
Nivolumab	117	48.1	93	48.4	24	34.3	8	28.6	4	26.7	20	51.3	7	38.9
pembrolizumab	26	10.7	18	9.4	8	11.4	–	–	–	–	–	–	–	–
Line of ICI														
1	11	4.5	11	5.7	0	0.0	–	–	–	–	–	–	–	–
2	140	57.6	135	70.3	5	7.1	24	85.7	1	6.7	34	87.2	–	–
3	42	17.3	29	15.1	13	18.6	4	14.3	3	20.0	5	12.8	7	38.9
4	40	16.5	15	7.8	25	35.7	0	0.0	5	33.3	–	–	6	33.3
5	19	7.8	2	1.0	17	24.3	0	0.0	3	20.0	–	–	3	16.7
6+	10	4.1	–	–	10	14.3	0	0.0	3	20.0	–	–	2	11.1
PD-L1 expression by IHC														
Negative	80	32.9	53	27.6	27	38.6	5	17.9	9	60.0	11	28.2	10	55.6
1~49%	102	42.0	82	42.7	20	28.6	14	50.0	5	33.3	19	48.7	4	22.2
≥50%	73	30.0	54	28.1	19	27.1	9	32.1	1	6.7	8	20.5	3	16.7
Unknown	7	2.9	3	1.6	4	5.7	–	–	–	–	1	2.6	1	5.6
Mutation														
*EGFR*	60	24.7	–	–	60	85.7	–	–	13	86.7	–	–	14	77.8
*ALK*	7	2.9	–	–	7	10.0	–	–	1	6.7	–	–	3	16.7
*ROS1*	3	1.2	–	–	3	4.3	–	–	1	6.7	–	–	1	5.6

CR, complete response; CyTOF, cytometry by time of flight; DCB, durable clinical benefit; LUAD, lung adenocarcinoma; LUSC, lung squamous cell carcinoma; MT, mutant type (*EGFR*, *ALK*-positive, or *ROS1* rearrangement); NDB, no durable clinical benefit; NSCLC, non-small cell lung cancer; PD, progression disease; PR, partial response; ROS1, rearrangement; SD, stable response; WT, wild-type.

PD-L1 expression patterns varied between the two groups (*P = 0.0965;* **Figure 1F**). MT patients exhibited a higher PD-L1 negativity, whereas WT patients more frequently had high PD-L1 expression levels (≥50%), though PD-L1 alone showed limited predictive value (AUC = 0.58, **Figure 1G**). Representative PD-L1 IHC images corresponding to each expression category are provided in **Supplementary Figure 2**.

As frontline anti-PD-1/PD-L1 monotherapy is typically reserved for patients with high PD-L1 expression and MT patients receive targeted therapies first-line, subsequent comparisons were restricted to second- or later-line recipients (≥2*L*, **Table 1**; **Supplementary Figure 3**), in whom survival outcomes remained significantly worse for MT patients.

### Comparison of major peripheral immune cell compositions in *EGFR*-WT and MT patients receiving anti-PD-1/PD-L1 immunotherapy

**Figure 2:**
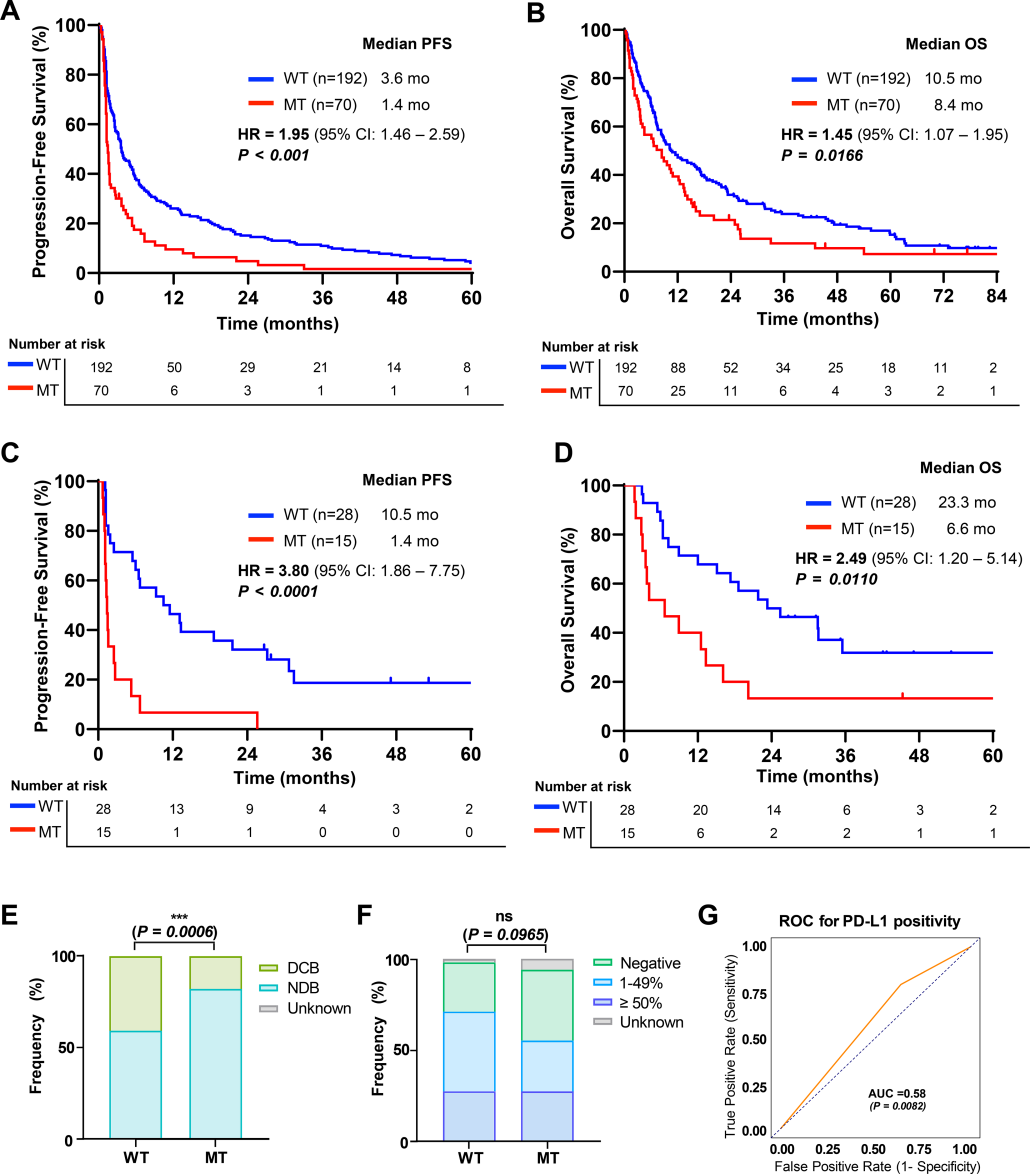
High-dimensional profiling of circulating major immune cell types. **(A)** Experimental design and analysis scheme for CyTOF. PBMCs were isolated from NSCLC patients at baseline and C2D1 of ICI treatment. **(B)** UMAP plot identifying eight major immune cell subsets from PBMCs, including CD4^+^ T cells, CD8^+^ T cells, double-negative T cells (DNT), Monocytes/Dendritic cells (Mono/DC), B cells, natural killer cells (NK), and basophils. **(C)** UMAP plots stratified by mutation status and time points(baseline and C2D1). **(D)** Heatmap showing the expression of lineage markers across the identified immune cell subsets. **(E)** Relative frequencies of the eight major immune cell subsets in individual patients (n=43). **(F)** Boxplots comparing the frequencies of immune cell subsets within CD45^+^ CD66b^−^ cells between WT and MT patients, both before and after ICI treatment. **(G)** Comparison of CD8^+^ T cell frequencies between WT and MT patients at baseline (*P = 0.0492*). **(H)** ROC curve for predicting treatment response based on the baseline frequency of CD8^+^ T cells (AUC = 0.634, *P = 0.0302*) (n = 43). *P < 0.05.

CyTOF analysis was performed on 86 PBMC samples collected at baseline and C2D1 from a prospective cohort of 43 NSCLC patients (WT: n=28; MT: n=15; **Figure 2A**), whose baseline demographics and clinical outcomes were consistent with those of the retrospective cohort (**Figures 1C, D**, **Table 1**). Individual tumor burden changes are visualized in **Supplementary Figure 4A**. The antibody panel was designed to identify major immune cell populations with a focus on T-cell subset phenotyping (**Supplementary Table 1**), given prior evidence linking anti-PD-1/PD-L1 resistance in NSCLC to circulating T-cell phenotypes. (23–25).

After gating on CD45^+^CD66b^−^ cells, we identified eight major immune cell clusters based on their canonical marker expression, including CD4^+^ T cells, CD8^+^ T cells, double-negative T cells (DNT), monocytes/dendritic cells (Mono/DC), B cells, natural killer cells (NK), and basophils, as displayed using uniform manifold approximation and projection (UMAP) analysis (**Figures 2B–D**). These clusters were derived from all 43 patients, without substantial bias toward any single patient (**Figure 2E**; **Supplementary Figure 5**).

Notably, WT patients exhibited significantly higher baseline CD8^+^ T cell frequencies and increased CD4^+^ T cells frequencies at C2D1 compared to MT patients (**Figure 2F**). Although differences in baseline CD8^+^ T cell frequencies between MT and WT were observed (*P = *0.0492; **Figure 2G**), their overall predictive value was limited (AUC = 0.634; **Figure 2H**). Additionally, CD4^+^ T cell frequencies at C2D1 differed significantly between WT and MT groups (*P = 0.0291*). While MT patients showed a trend toward decreased CD4^+^ T cell and increased CD8^+^ T cells, the change was not statistically significant (*P = 0.0681*).

Collectively, these findings suggest that while baseline CD8+ T-cell differences offer only modest predictive power, specific CD8^+^ T-cell clusters in WT patients may serve as more robust indicators of anti PD-1/PD-L1 response. Furthermore, the observed trend in CD4^+^ T-cell dynamics suggests potential distinct immunological patterns during therapy.

### Baseline CXCR3^+^ CD127^+^ effector CD8^+^ T cells are elevated in WT and predict immunotherapy response

**Figure 3:**
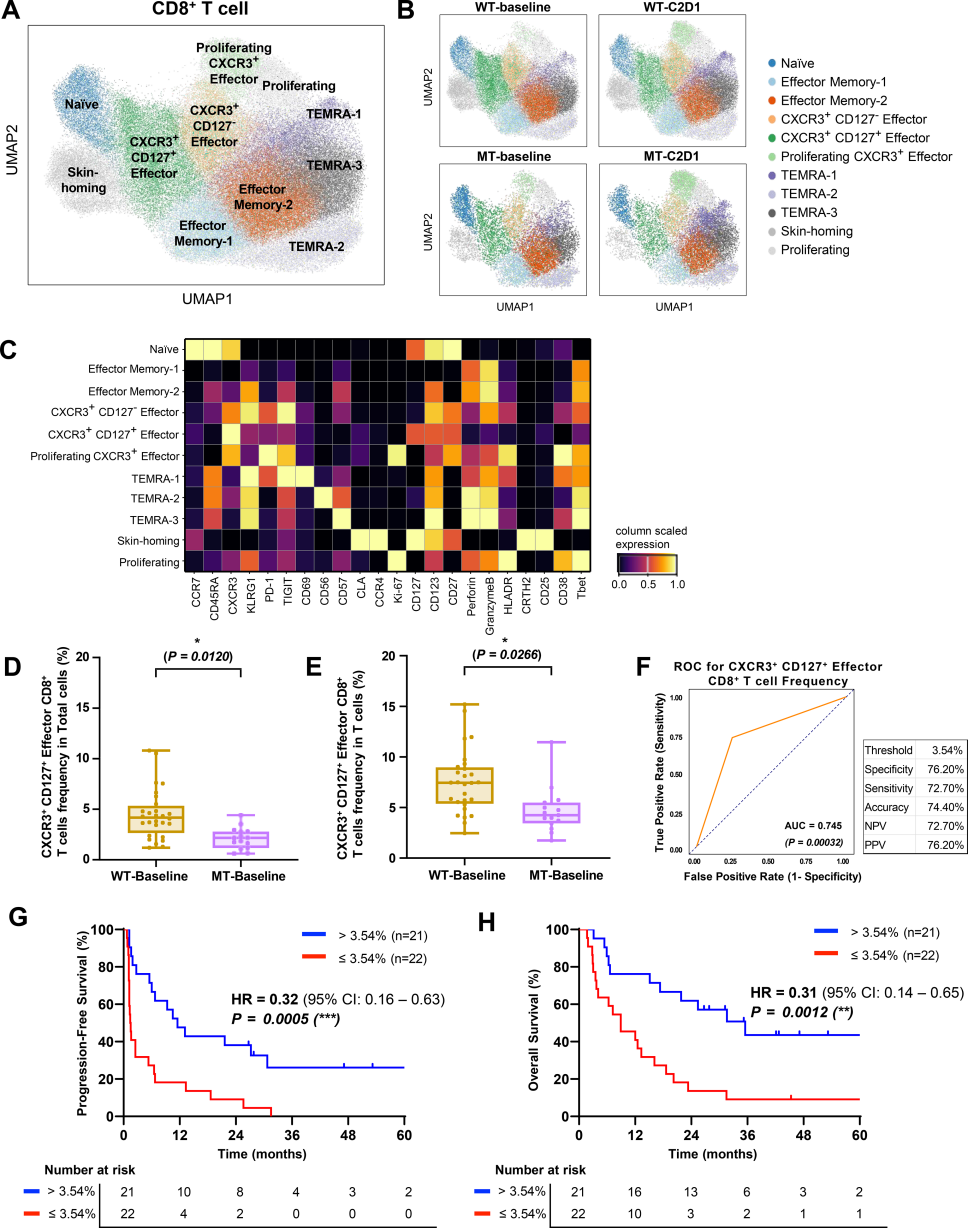
CXCR3^+^ CD127^+^ effector CD8^+^ T cells are associated with improved survival in WT NSCLC patients treated with immune checkpoint inhibitors. **(A–C)** High-dimensional CyTOF analysis of circulating CD8^+^ T cells in peripheral blood mononuclear cells (PBMCs). **(A)** UMAP visualization identifying 11 distinct CD8^+^ T-cell subsets. **(B)** UMAP plot showing the distribution of the 11 CD8^+^ T-cell subsets stratified by mutation status and sampling time. **(C)** Heat map depicting the expression of key phenotypic markers across the CD8^+^ T-cell subsets. **(D, E)** Comparison of the frequency of CXCR3^+^ CD127^+^ effector CD8^+^ T cells before immune checkpoint inhibitor (ICI) treatment in the discovery cohort, expressed as a proportion of total cells [**(D)**, *P = 0.0120*] and as a proportion of total T cells [**(E)**, *P = 0.0266*]. **(F)** Receiver operating characteristic (ROC) curve evaluating the predictive performance of the frequency of CXCR3^+^ CD127^+^ effector CD8^+^ T cells (relative to total cells) for treatment response in the discovery cohort (AUC = 0.745; *P = 0.00032*). **(G, H)** Kaplan–Meier curves for progression-free survival **(G)** and overall survival **(H)** in patients stratified by the baseline frequency of CXCR3^+^ CD127^+^ effector CD8^+^ T cells relative to total cells prior to ICI treatment. *P < 0.05; **P < 0.01; ***P < 0.001.

We therefore focused on CD8^+^ T cells, identifying 11 distinct CD8^+^ T cell subset clusters by UMAP dimensionality reduction (**Figures 3A–C**; **Supplementary Figure 6A**). Prior to anti-PD-1/PD-L1 treatment, only the CXCR3^+^ CD127^+^ effector CD8^+^ T cell clusters significantly differed between WT and MT groups (**Supplementary Figure 6A**). Specifically, the WT group showed a significantly higher baseline frequency of CXCR3^+^ CD127^+^ Effector CD8^+^ T cells, both as a proportion of total cells and of total T cells, compared to the MT group (*P = 0.0120*; **Figure 3D**, *P = 0.0266*; **Figure 3E**). This difference remained significant after adjustment for potential confounders including sex (or smoking history), age, and histological type in multivariate linear regression analyses (**Supplementary Table 4**). Comparison of CXCR3 and CD127 marker expression levels revealed no significant baseline differences between groups (**Supplementary Figure 6B**), suggesting that the differences between the groups are primarily attributable to the abundance of CXCR3^+^ CD127^+^ effector CD8^+^ T cells rather than variations in quantitative marker expression.

Receiver operating characteristic (ROC) curve analysis demonstrated that PD-L1 positivity nor the overall frequency of CD8^+^ T cells relative to total cells (**Figures 1G**, **2H**) reached statistical significance for predicting anti-PD-1/PD-L1 treatment response. In contrast, the frequency of CXCR3^+^ CD127^+^ effector CD8+ T cell within total cells was strongly correlated with treatment response (AUC: 0.745; **Figure 3F**). At a cutoff threshold of 3.54%, patients with higher baseline CXCR3^+^ CD127^+^ effector CD8^+^ T cells frequencies, measured as a percentage of total cells, exhibited significantly improved PFS (HR = 0.32, 95% CI = 0.16-0.63, *P = 0.0005*; **Figure 3G**) and OS (HR = 0.31, 95% CI = 0.14–0.65, *P = 0.0012*; **Figure 3H**).

**Figure 4:**
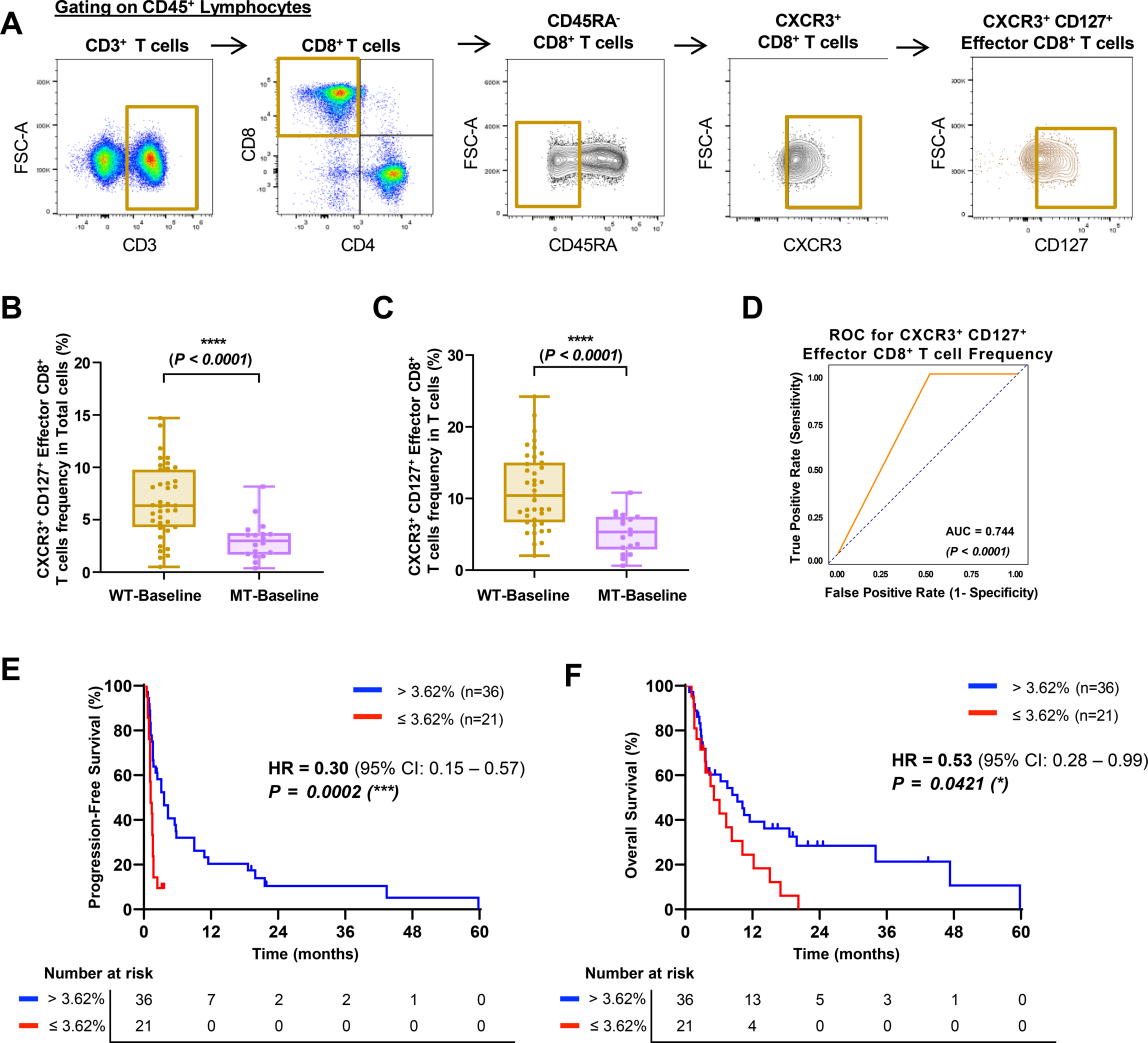
Flow cytometry–based quantification of CXCR3^+^ CD127^+^ effector CD8^+^ T cells predicts improved immunotherapeutic response in independent WT NSCLC cohorts. **(A)** Representative flow cytometry gating strategy used to identify CXCR3^+^ CD127^+^ effector CD8^+^ T cells, quantified relative to total cells. **(B, C)** Comparison of the frequency of CXCR3^+^ CD127^+^ effector CD8^+^ T cells before immune checkpoint inhibitor (ICI) treatment in the validation cohort, expressed as a proportion of total cells [**(B)**, *P < 0.0001*] and as a proportion of total T cells [**(C)**, *P < 0.0001*]. **(D)** Receiver operating characteristic (ROC) curve evaluating the predictive performance of the frequency of CXCR3^+^ CD127^+^ effector CD8^+^ T cells relative to total cells for treatment response in the validation cohort (AUC = 0.744; *P < 0.0001*). **(E, F)** Kaplan–Meier curves for progression-free survival **(E)** and overall survival **(F)** in patients stratified by the frequency of CXCR3^+^ CD127^+^ effector CD8^+^ T cells relative to total cells before ICI treatment. *P < 0.05; ***P < 0.001; ****P < 0.0001.

To validate these findings, we conducted a separate flow cytometry study in an independent cohort (WT: n=39; MT: n=18) using PBMCs collected at the same time points as in the discovery cohort (**Supplementary Table 2**). Based on the CyTOF-defined phenotype (**Figure 3C**; **Supplementary Table 5**), we characterized CXCR3^+^ CD127^+^ effector CD8^+^ T cells by gating on CD45RA^-^, CXCR3^+^, and CD127^+^ expressions (**Figure 4A**).

Consistent with the CyTOF analysis, the WT patient group in the validation cohort exhibited a significantly higher baseline frequency of CXCR3^+^ CD127^+^ effector CD8^+^ T cells, compared to the MT group, both as a percentage of total cells and of T cells, (both *P < 0.0001*; **Figures 4B, C**). ROC curve analysis further confirmed a strong correlation between CXCR3^+^ CD127^+^ effector CD8^+^ T cell frequency and anti-PD-1/PD-L1 response (AUC = 0.744; **Figure 4D**). Using a threshold of 3.62%, patients with baseline CXCR3^+^ CD127^+^ effector CD8^+^ T cells frequencies above this cut-off demonstrated improved PFS (HR = 0.30, 95% CI = 0.15-0.57, *P = 0.0002*; **Figure 4E**) and OS (HR = 0.53, 95% CI = 0.28-0.99, *P = 0.0421*; **Figure 4F**). The corresponding waterfall plot depicting individual changes in tumor burden within the validation cohort is presented in **Supplementary Figure 4B**.

Overall, these findings indicate baseline CXCR3^+^ CD127^+^ effector CD8^+^ T cell frequency as a candidate predictive biomarker of response to anti-PD-1/PD-L1, outperforming traditional biomarkers such as PD-L1 expression and overall CD8^+^ T cell frequency in predictive accuracy.

### Distinct reduction dynamics of circulating CD27^+^ PD-1^-^ effector memory CD4^+^ T cells in MT NSCLC patients correlate with poor prognosis

Beyond baseline prognostic markers, changes in peripheral immune cell dynamics during treatment have been linked to clinical benefits in cancer patients (26). We therefore compared immune cell dynamics in PBMCs at baseline and C2D1 between groups, normalizing data as fold changes to account for inter-individual variability. CXCR3^+^ CD127^+^ effector CD8^+^ T cells declined significantly after treatment in the WT group (*P = 0.0017*; **Supplementary Figure 6C**, *P = 0.0006*; **Supplementary Figure 6E**), but not in the MT group (**Supplementary Figures 6D, E**), a pattern replicated in the validation cohort (**Supplementary Figures 7A–C**).

Among the major cell types, only the CD4^+^ T cell cluster exhibited a significantly different proportion in C2D1, whereas no difference was observed at baseline between the WT and MT groups (**Figure 2F**). Furthermore, a correlation analysis of marker expression within the CD4^+^ T cell population revealed a cytotoxic module characterized by CD11c, CD57, KLRGL1, Perforin, Tbet, and Granzyme B. Notably, this cytotoxic module was downregulated in post-treatment samples in the MT but not WT group (**Supplementary Figures 8A, B**), prompting further analysis of CD4^+^ T cell subsets.

**Figure 5:**
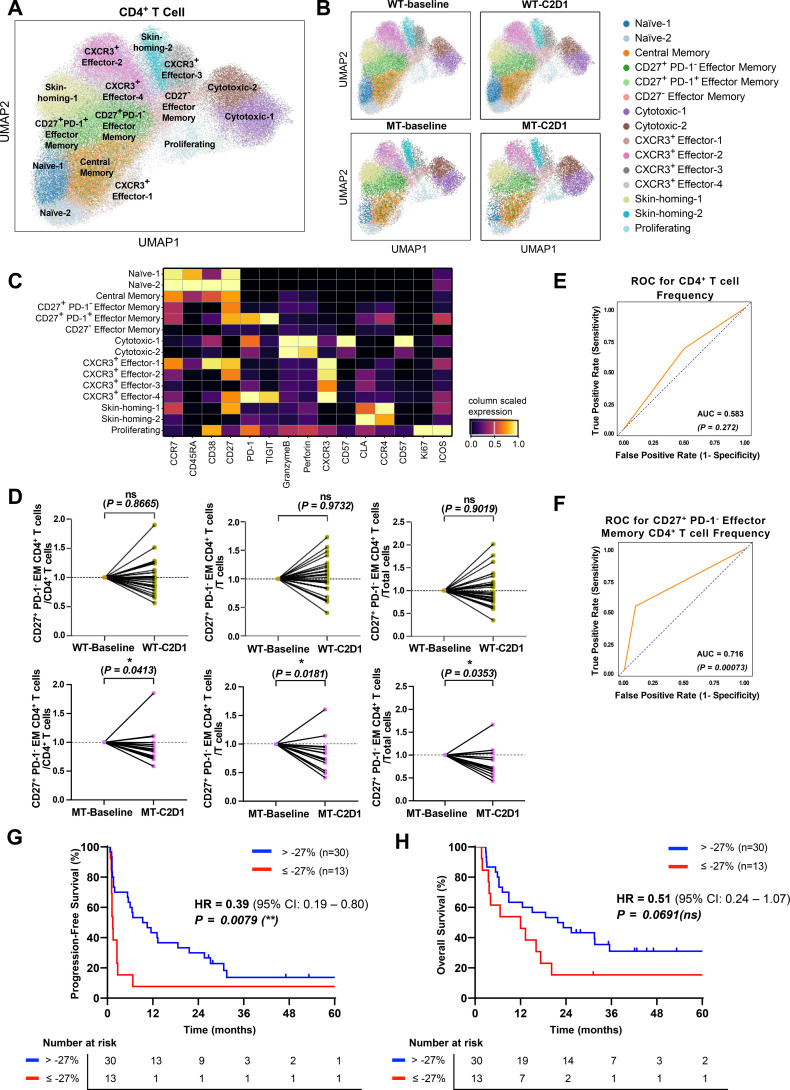
Dynamic reduction of CD27^+^ PD-1⁻ effector memory CD4^+^ T cells following ICI treatment is associated with poor immunotherapeutic response in MT NSCLC patients. **(A–C)** High-dimensional CyTOF analysis of circulating CD4^+^ T cells in peripheral blood mononuclear cells (PBMCs). **(A)** UMAP visualization identifying 15 distinct CD4^+^ T-cell subsets. **(B)** UMAP plot showing the distribution of the 15 CD4^+^ T-cell subsets stratified by mutation status and sampling time. **(C)** Heat map depicting the expression of key phenotypic markers across the CD4^+^ T-cell subsets. **(D)** Dynamic changes in the frequency of CD27^+^ PD-1⁻ effector memory (EM) CD4^+^ T cells, expressed relative to CD4^+^ T cells, total T cells, and total cells, in WT and MT patients before and after immune checkpoint inhibitor (ICI) treatment. **(E, F)** Receiver operating characteristic (ROC) curves evaluating the predictive performance for treatment response using the frequency of total CD4^+^ T cells relative to total cells [**(E)**, AUC = 0.583; *P = 0.272*] and the frequency of CD27^+^ PD-1⁻ effector memory CD4^+^ T cells relative to total cells [**(F)**, AUC = 0.716; *P = 0.00073*]. **(G, H)** Kaplan–Meier curves for progression-free survival (PFS) **(G)** and overall survival (OS) **(H)** in patients stratified by the post-treatment reduction in the frequency of CD27^+^ PD-1⁻ effector memory CD4^+^ T cells relative to total cells following ICI treatment. *P < 0.05; **P < 0.01; ns, not significant.

UMAP analysis identified 15 CD4^+^ T cell clusters (**Figures 5A–C**; **Supplementary Figure 9A**). Among these clusters, only CD27^+^PD-1^-^effector memory CD4^+^ T cell (excluding skin-homing T cells) showed a trend toward significance in dynamic differences between WT and MT patients (*P=0.0504*; **Supplementary Figure 9A**).

Specifically, the frequency of CD27^+^ PD-1^-^ effector memory CD4+ T cells (relative to CD4^+^ T cells, total T cells, and total cells) was significantly reduced in MT patients following anti-PD-1/PD-L1 treatment, while no significant reduction was observed in WT patients (**Figure 5D**). No differences in CD27 expression were detected between the WT and MT groups at either baseline or C2D1 (**Supplementary Figure 9B**).

ROC curve analysis indicated that changes in overall CD4^+^ T cell frequency did not significantly predict anti-PD-1/PD-L1 response (AUC = 0.583; **Figure 5E**). However, a ≥27% reduction(fold change of ≤-27%) in CD27^+^ PD-1^-^effector memory CD4^+^ T cell frequency (relative to total cells) from baseline to C2D1 was associated with poor treatment response (AUC = 0.716; **Figure 5F**). Patients with this less than 27% reduction (> -27%) showed superior PFS (HR = 0.39, 95% CI = 0.19-0.80, *P = 0.0079*; **Figure 5G**) and a trend toward improved OS (HR = 0.51, 95% CI = 0.24-1.07, *P = 0.0691*; **Figure 5H**) compared to those with ≥27% reduction (≤-27%).

**Figure 6:**
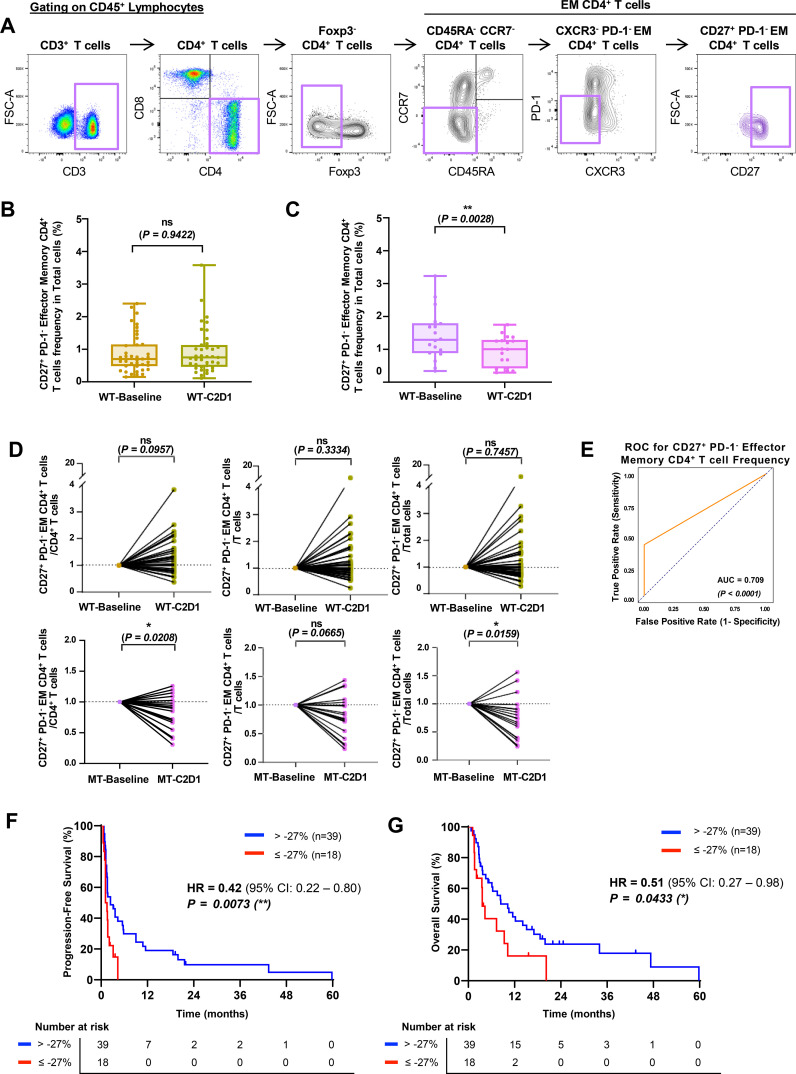
Flow cytometry–based dynamics of CD27^+^ PD-1⁻ effector memory CD4^+^ T cells are associated with poor immunotherapeutic response in MT NSCLC patients. **(A)** Representative flow cytometry gating strategy used to identify CD27^+^ PD-1⁻ effector memory CD4^+^ T cells, quantified relative to total cells. **(B, C)** Comparison of the frequency of CD27^+^ PD-1⁻ effector memory CD4^+^ T cells in the validation cohort, expressed as a proportion of total cells [**(B)**, *P = 0.9422*] and as a proportion of total T cells (**C**, *P = 0.0028*), before and after immune checkpoint inhibitor (ICI) treatment. **(D)** Dynamic changes in the frequency of CD27^+^ PD-1⁻ effector memory CD4^+^ T cells expressed relative to CD4^+^ T cells, total T cells, and total cells, stratified by mutation status (WT vs MT) before and after ICI treatment in the validation cohort. **(E)** Receiver operating characteristic (ROC) curve evaluating the predictive performance of the CD27^+^ PD-1⁻ effector memory CD4^+^ T cells–to–total cells ratio for treatment response in the validation cohort (AUC = 0.709; *P <0.0001*). **(F, G)** Kaplan–Meier curves for progression-free survival **(F)** and overall survival **(G)** in patients stratified by the post-treatment reduction in the frequency of CD27^+^ PD-1⁻ effector memory CD4^+^ T cells relative to total cells following ICI treatment. *P < 0.05; **P < 0.01; ns, not significant.

Validation was performed in the same cohort (n = 57) used for CD8^+^ T cell analyses. CD27^+^ PD-1^-^ effector memory CD4^+^ T cells were defined using the following markers: CD45RA^-^, CCR7^-^, PD-1^-^, CXCR3^-^, and CD27^+^ per the CyTOF-defined phenotype (**Figures 5C**, **6A****;** **Supplementary Table 6**). Consistent with discovery cohort findings, the frequency of CD27^+^PD-1^-^effector memory CD4^+^ T cell relative to total cells decreased significantly after anti-PD-1/PD-L1 in MT patients but not in WT patients (*P = 0.9422*; **Figure 6B**, *P = 0.0028*; **Figure 6C**). Similar trends emerged when normalizing CD27^+^ PD-1^-^effector memory CD4^+^ T cell dynamics to CD4^+^ T cells, total T cells, or total cells (**Figure 6D**).

ROC curve analysis confirmed this correlation (AUC = 0.709; **Figure 6E**) and independently identified a ≥27% reduction threshold(fold change of ≤-27%), consistent with the discovery cohort, as a predictor of poor treatment response. In the validation cohort, patients with a <27% reduction (fold change of >-27%) in CD27^+^ PD-1^-^effector memory CD4^+^ T cells demonstrated superior PFS (HR = 0.42, 95% CI = 0.22-0.80, *P = 0.0073*; **Figure 6F**) and OS (HR = 0.51, 95% CI = 0.27-0.98, *P = 0.0433*; **Figure 6G**) compared to those with a ≥27% reduction (fold change of ≤-27%).

### Dynamics of CD27^+^ PD-1^-^ effector memory CD4^+^ T cells in MT patients correlate with decreased conventional dendritic cells dynamics during anti-PD-1/PD-L1 treatment

Our study shows CD27^+^ PD-1^-^ effector memory CD4^+^ T cells in PBMCs is associated with poor prognosis in MT-NSCLC. Given that dendritic cells (DCs) play a crucial role in antigen presentation (27), we investigated whether the reduction in CD27^+^ PD-1^-^ effector memory CD4^+^ T cells was associated with DC dynamics as well.

To elucidate the mechanism, we characterized the phenotypes of 4 myeloid cell subsets (**Supplementary Figures 10A–C**), of which conventional dendritic cells (cDC) and plasmacytoid dendritic cells (pDC) showed a reduction in the MT group after anti-PD-1/PD-L1 (**Supplementary Figures 10D**, **11**). However, since pDCs constituted less than 1% of detected cells, they were excluded from further analysis to minimize potential bias.

HLA-DR expression on cDCs did not differ between the WT and MT groups (**Supplementary Figure 10E**), indicating that the observed differences primarily reflect a loss in cDC abundance rather than altered functional marker expression. ROC analysis identified a less than 57.5% reduction in cDCs relative to total cells after anti-PD-1/PD-L1 as a predictor of response (AUC = 0.711; **Supplementary Figure 10F**). Patients exhibiting less than 57.5% cDC reduction (>-57.5%) demonstrated superior PFS (HR = 0.22, 95% CI = 0.08-0.55, *P = 0.0004*; **Supplementary Figure 10G**) and OS (HR = 0.36, 95% CI = 0.15-0.85, *P = 0.0149*; **Supplementary Figure 10H**), compared to those with ≥57.5% reduction (≤-57.5%).

To confirm the association between cDC reduction and the loss of CD27^+^ PD-1^-^ effector memory CD4^+^ T cells, we examined their correlation, which revealed a significant positive relationship (r = 0.53, P = 0.00029; **Supplementary Figure 10I**). This suggests that the decline in CD27^+^ PD-1^-^ effector memory CD4^+^ T cells is consistent with the reduction in cDCs.

## Discussion

Our study highlights distinct peripheral immune cell profiles that differentiate responses to PD-1/PD-L1 ICIs in WT and MT NSCLC patients. By employing high-dimensional immune profiling, we identified key biomarkers predictive of treatment outcomes and critical immune mechanisms that contribute to the limited efficacy of ICIs in MT-NSCLC.

A significant finding of this study is the elevated baseline frequency of CXCR3^+^ CD127^+^ effector CD8^+^ T cells in WT-NSCLC patients, which was predictive of favorable responses to PD-1/PD-L1 blockade. Higher baseline frequencies of this subset in WT patients (*P* = 0.0120) were associated with prolonged progression-free survival (PFS) and overall survival (OS) (AUC = 0.745). Notably, this phenomenon was predominantly observed in WT patients and demonstrated superior performance compared to traditional PD-L1 biomarkers in predicting anti-PD-1/PD-L1 response.

CXCR3 is a CXC subgroup receptor present on activated T cells, B cells, and NK cells, which selectively binds CXCL9/10/11 to regulate tumor immunity, migration, and immune activation (28–30). The CXCL9/10/11-CXCR3 axis plays a crucial role in T cell recruitment and immune cell infiltration into tumor sites in NSCLC (31), contributing to anti-PD-1/PD-L1 therapeutic efficacy, as seen in other cancers (29, 32, 33). Additionally, effector CD8^+^ T cells can be categorized based on CD127 and KLRG1 expression (34, 35). CD127 (IL7Rα) functions as a marker of the classic long-lived memory T cells (36, 37), and CD127^+^ KLRG1^-^ CD8^+^ T cells have been identified as memory precursor effector T cells (38). An increased number of intra-tumoral CD127^+^ KLRG1^-^ CD8^+^ T cells has been associated with better prognosis in both follicular lymphoma and NSCLC (36, 39). Consistent with prior reports showing higher CXCL9/10/11 levels in the tumor microenvironment (TME) and increased intra-tumoral CD8^+^ T cells in WT-NSCLC (40, 41), our data demonstrated that CXCR3^+^ CD127^+^ effector CD8^+^ T cells were elevated at baseline and significantly declined following anti-PD-/PD-L1 treatment in WT patients. In contrast, MT patients showed neither baseline elevation nor a significant post-treatment decline in this population.

This subset may therefore serve as a peripheral indicator of pre-existing immune activation that ICIs can further augment. Conversely, the lower frequency of these cells in MT patients may reflect a less favorable baseline immune environment which could be associated with their poorer clinical outcomes. Future studies should explore the mechanistic roles of CXCR3 and CD127 in tumor immunity and investigate whether targeting these markers could enhance anti-tumor responses.

Moreover, MT patients showed a significant decline of CD27^+^ PD-1^-^ effector memory CD4^+^ T cells and cDCs, which are associated with poor anti-PD-1/PD-L1 prognosis. The Th1- and Th17-promoting cytokines secreted by activated cDCs play a crucial role in immune response (42). CD27, a TNF receptor family member expressed on T cells, supports the immune responses by enhancing T cell activation, promoting cell survival, and facilitating differentiation into antigen-specific cytotoxic and memory T cells (43–45). As cDCs are essential for effective T cell priming through antigen presentation and co-stimulation, the observed reduction in cDC abundance is possibly associated with decreased CD27^+^ PD-1^-^ effector memory CD4^+^ T cell frequency, suggesting potential impairment of adaptive immunity in MT patients. These findings raise the possibility that disruption of the T-cell–dendritic cell axis may contribute to limiting the effectiveness of anti-PD-1/PD-L1 in MT patients, though direct mechanistic evidence remains to be established.

Notably, although CD27^+^ PD-1⁻ effector memory CD4^+^ T cells are not direct targets of PD-1/PD-L1 blockade, their dynamic reduction may reflect broader impairment of adaptive immune coordination in MT patients. Accumulating evidence suggests that systemic CD4^+^ T cell immunity plays an important helper role in sustaining effective CD8^+^ T cell responses during checkpoint inhibition (46, 47). In this context, the observed decline in this population may serve as a surrogate marker of impaired cDC–CD4^+^–CD8^+^ T cell crosstalk, rather than a direct consequence of PD-1/PD-L1 blockade.

Several limitations of this study should be acknowledged. Although patients were drawn from prospectively collected biomarker clinical trials, selection for immune profiling was further conditioned on PBMC sample quality and representation across mutation status and clinical response groups. While this approach was intended to minimize systematic bias, the possibility of residual selection bias cannot be entirely excluded. Of note, the difference in prior lines of therapy between WT and MT groups reflects standard-of-care sequencing in oncogenic mutation-positive NSCLC, but may have introduced residual confounding despite multivariable adjustment for relevant covariates.

Additionally, PD-L1 expression data were obtained from routine clinical records using different IHC assays (SP263, 22C3, and SP142). Given the known inter-assay variability across these platforms, this may have introduced inconsistency in PD-L1 scoring, which could have influenced the observed limited predictive value of PD-L1 expression in our cohort. Lastly, the relatively small sample size, particularly in the MT subgroup of the discovery cohort (n=15), suggests that the ROC-derived cut-off values may be dataset-specific. Nonetheless, the cut-off of ≥3.54% was independently replicated at ≥3.62% in the validation cohort, offering preliminary support for its generalizability, and encourages further validation in larger, prospective cohorts.

In conclusion, our study demonstrates that CXCR3^+^ CD127^+^ effector CD8^+^ T cells in PBMCs may serve as promising biomarkers for predicting anti-PD-1/PD-L1 responses in WT-NSCLC. Additionally, an early decline in CD27^+^ PD-1^-^ effector memory CD4^+^ T cells and cDCs is associated with poor prognosis in MT-NSCLC. Our findings highlight the feasibility of peripheral blood immune monitoring in guiding ICI therapy and improving patient stratification in WT and MT NSCLC patients. These insights provide a basis for improved patient stratification and highlight therapeutic strategies aimed at restoring cDC function and enhancing effector T cell responses to improve immunotherapy outcomes in MT-NSCLC patients.

## Data Availability

The original contributions presented in the study are included in the article/**Supplementary material**. Further inquiries can be directed to the corresponding author/s.
