# Supplementary material for: High-dimensional immune profiling of peripheral blood identifies immune correlates of anti-PD-1/PD-L1 resistance in oncogenic driver mutation-positive NSCLC
Source: Front Immunol. 2026 May 29;17:1829816. doi: 10.3389/fimmu.2026.1829816 (PMC13260270; doi:10.3389/fimmu.2026.1829816)
Supplement: Supplementary file 1 [file DataSheet1.pdf]

Supplementary Figure 1

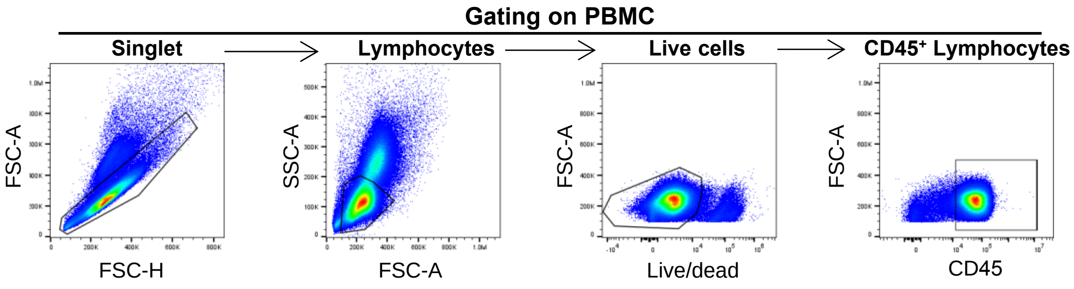

Supplementary Figure 1. Gating strategy on PBMC
A. Data normalization across all PBMC samples was performed by randomly down-sampling to 50,000 CD45$^+$ lymphocytes.

Supplementary Figure 2

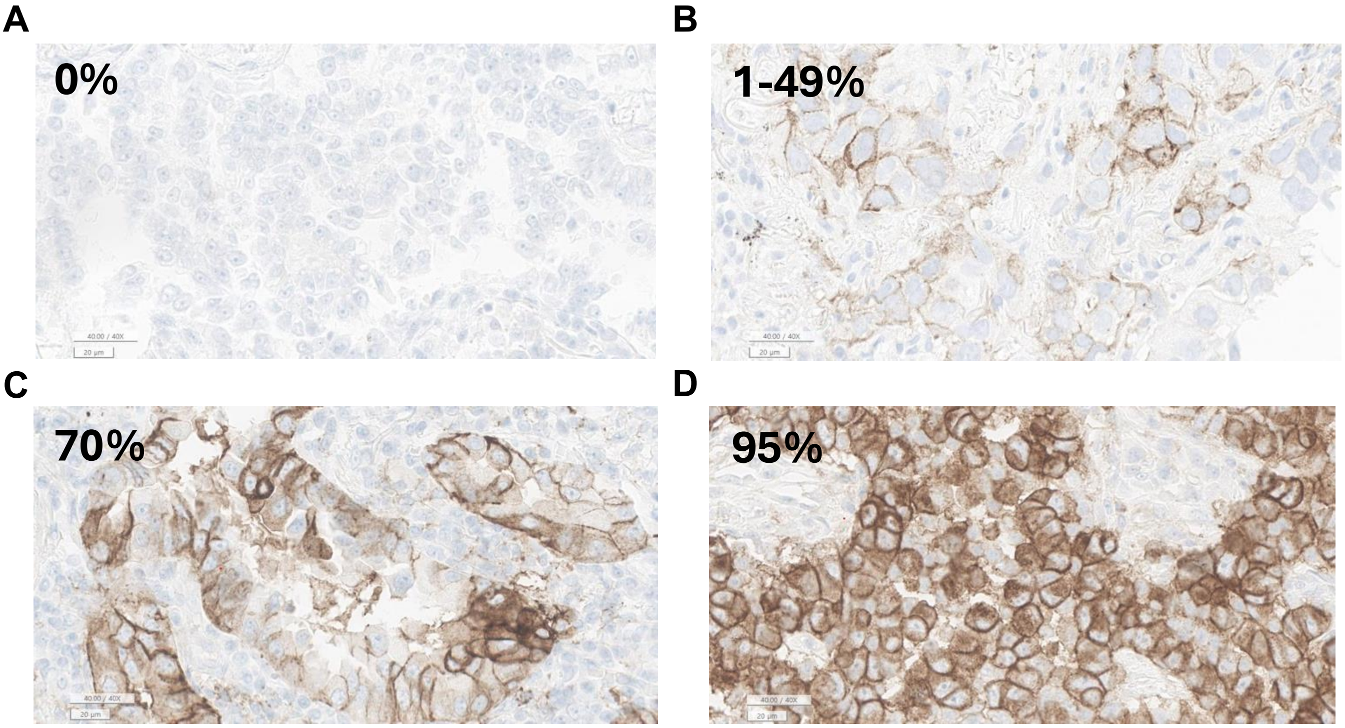

Supplementary Figure 2. Representative PD-L1 immunohistochemistry (IHC) images across different expression categories.

A-D. Representative PD-L1 IHC images from NSCLC tumor specimens corresponding to various expression categories: (A) negative (0%), (B) 1–49%, and (C-D) ≥ 50% (70% and 95%). Brown chromogenic staining indicates PD-L1 expression on tumor cells. Images were acquired at 40× magnification; scale bar = 20 μm.

Supplementary Figure 3

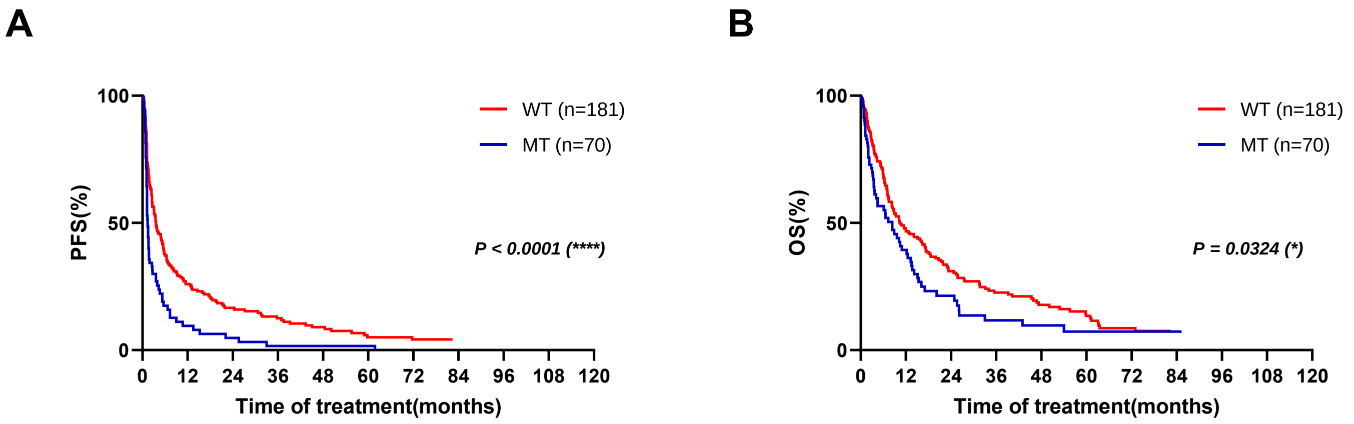

Supplementary Figure 3. Clinical outcomes of ICI in second- and further-line treatment settings

A-B. Second- or later-line ICI retrospective cohort and discovery cohort by (A) PFS and (B) OS of patients according to the mutation status.

Supplementary Figure 4

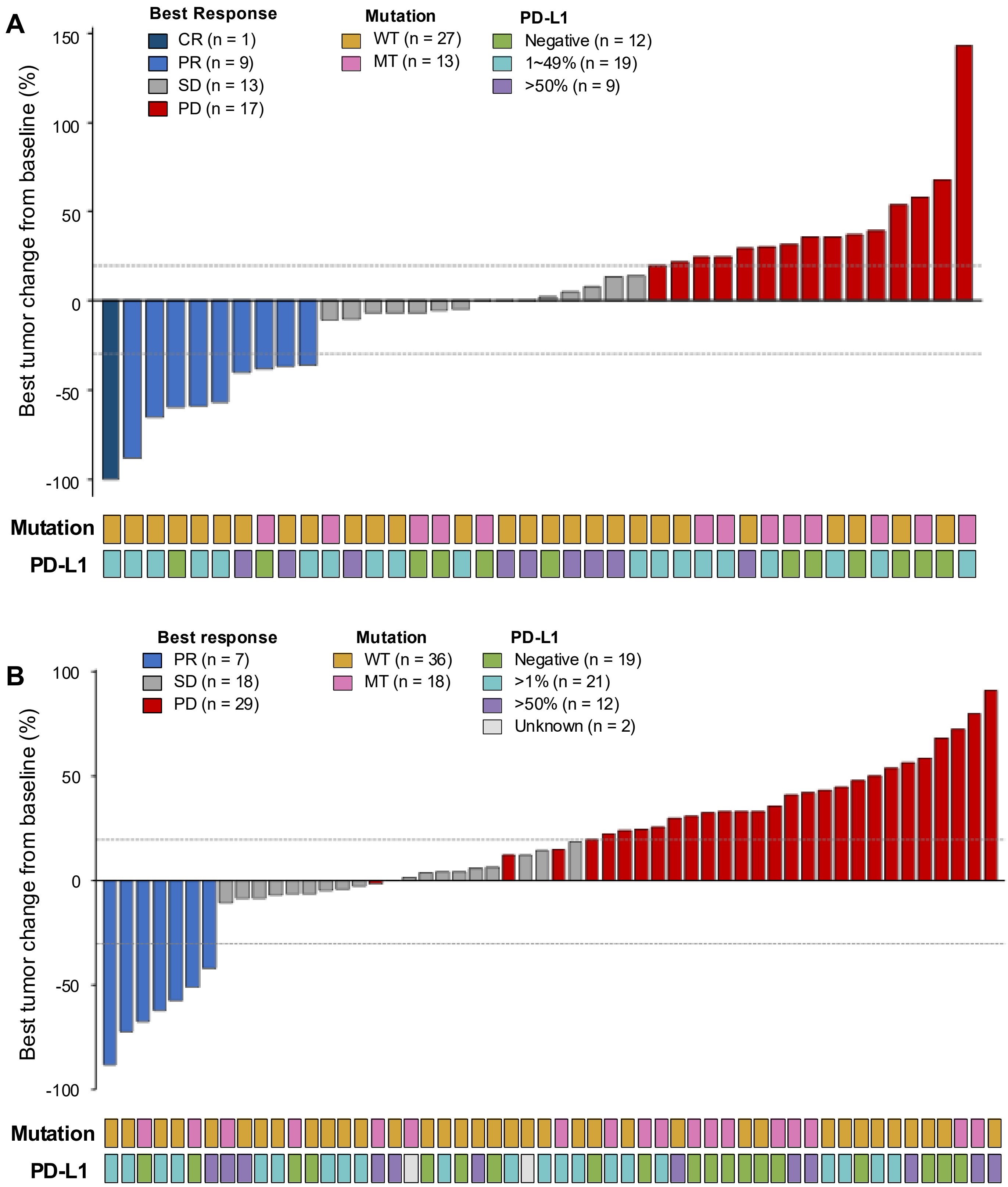

Supplementary Figure 4. Waterfall plots depicting best tumor response to anti-PD-1/PD-L1 monotherapy in the discovery (A) and validation (B) cohorts.
Each bar represents the maximum percent change in tumor burden from baseline per individual patient. Bars are colored according to best response category (CR, complete response; PR, partial response; SD, stable disease; PD, progressive disease). Mutation status (WT, wild-type; MT, mutant-type) and PD-L1 expression levels are indicated by colored tiles below each bar. Dotted lines indicate +20% and −30% thresholds per RECIST v1.1 criteria.

Supplementary Figure 5

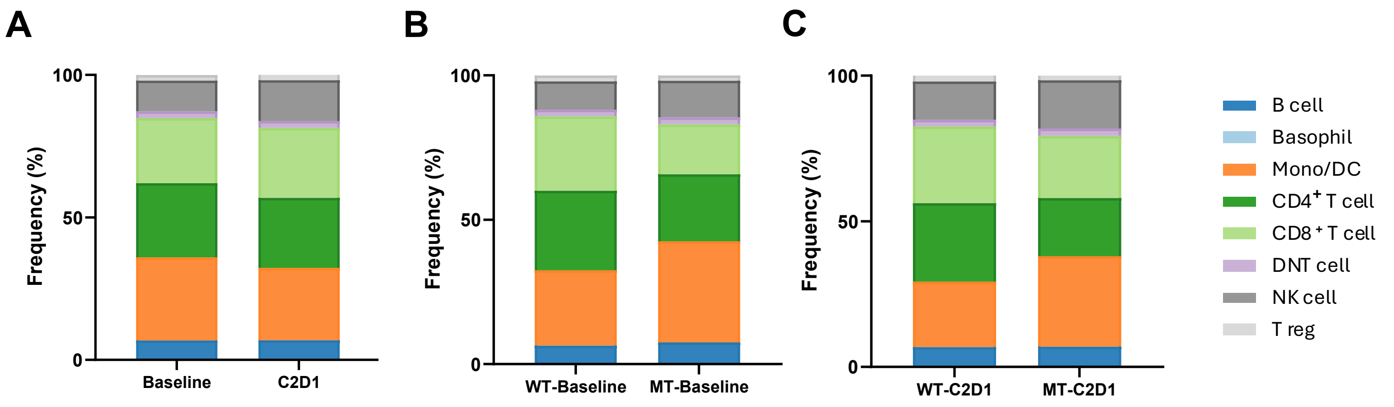

Supplementary Figure 5. Composition of circulating major immune cell types before and after ICI

A-C. Relative frequencies of major immune cell subsets in PBMCs from the (A) discovery cohort, (B) WT and MT groups at baseline, and (C) WT and MT groups at C2D1.

Supplementary Figure 6

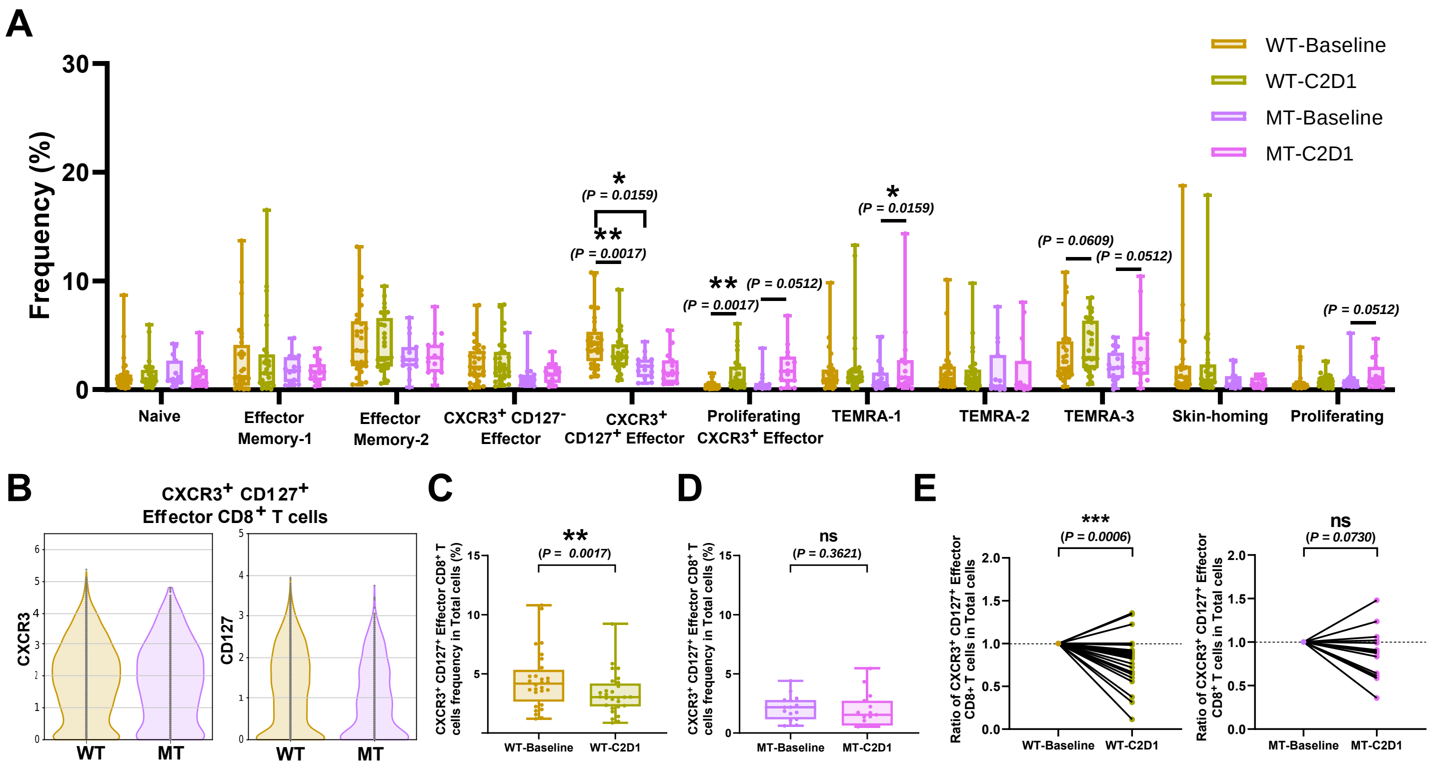

Supplementary Figure 6. Dynamic changes in the composition of CD8⁺ T cells before and after ICI

A. Boxplots demonstrating the frequencies of the 11 CD8⁺ T cell subsets in WT and MT patients before and after ICI. **B.** Violin plots for CXCR3⁺ CD127⁺ effector CD8⁺ T cells showing marker expression for CXCR3 and CD127 at baseline in WT and MT patients. **C-D.** CXCR3⁺CD127⁺ effector CD8⁺ T cells frequency relative to total cells (*P=0.0017*) in (C) WT patients and (D) MT patients (*P=0.3621*) before and after ICI in the validation cohort. **E.** Dynamic changes in CXCR3⁺ CD127⁺ effector CD8⁺ T cells ratio among the total cells in WT and MT patients before and after ICI in the validation cohort.

Supplementary Figure 7

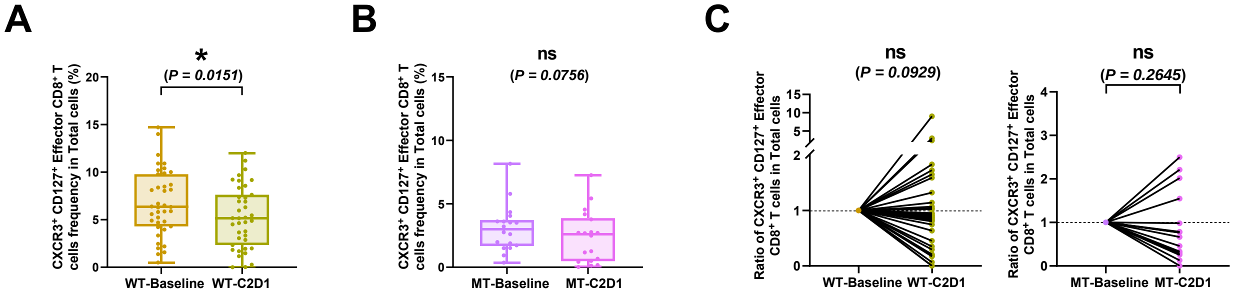

Supplementary Figure 7. Dynamic changes in CXCR3$^+$CD127$^+$ effector CD8+ T cells composition before and after ICI in the validation cohort

A. CXCR3$^+$CD127$^+$ effector CD8$^+$ T cells frequency relative to total cells (*P=0.0151*) in WT patients. **B.** CXCR3$^+$CD127$^+$ effector CD8$^+$ T cells frequency relative to T cells (*P=0.0756*) in MT patients before and after ICI in the validation cohort. **C.** Dynamic changes in the CXCR3$^+$CD127$^+$ effector CD8$^+$ T cells ratio among total cells in WT and MT patients before and after ICI in the validation cohort.

Supplementary Figure 8

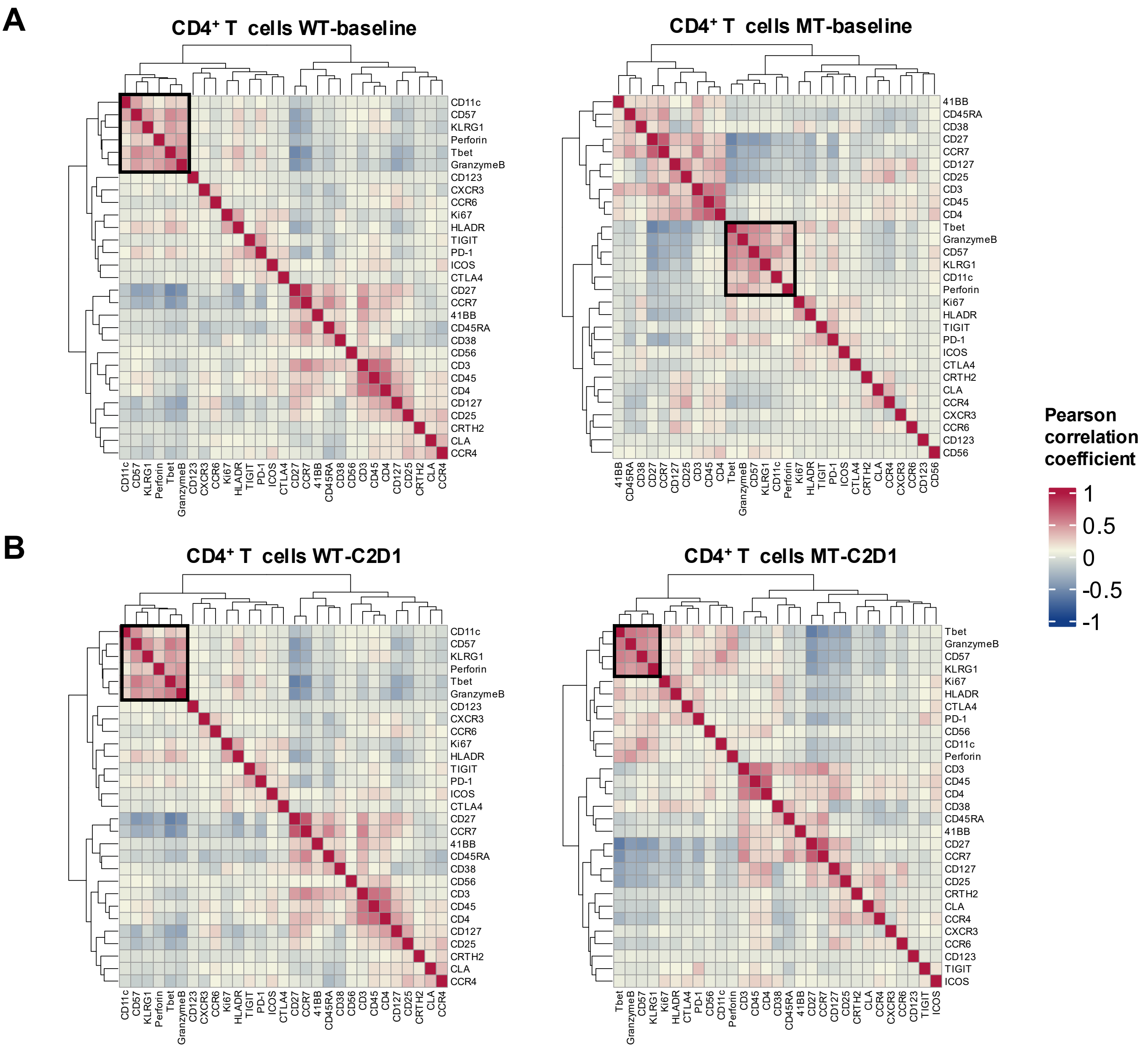

Supplementary Figure 8. Correlation of marker expression within the CD4+ T cells before and after ICI

A. Pearson correlation coefficient (PCC) heatmap matrix analysis of CyTOF markers in the WT and MT groups at baseline. **B.** PCC heatmap matrix analysis of CyTOF markers in the WT and MT groups at C2D1.

Supplementary Figure 9

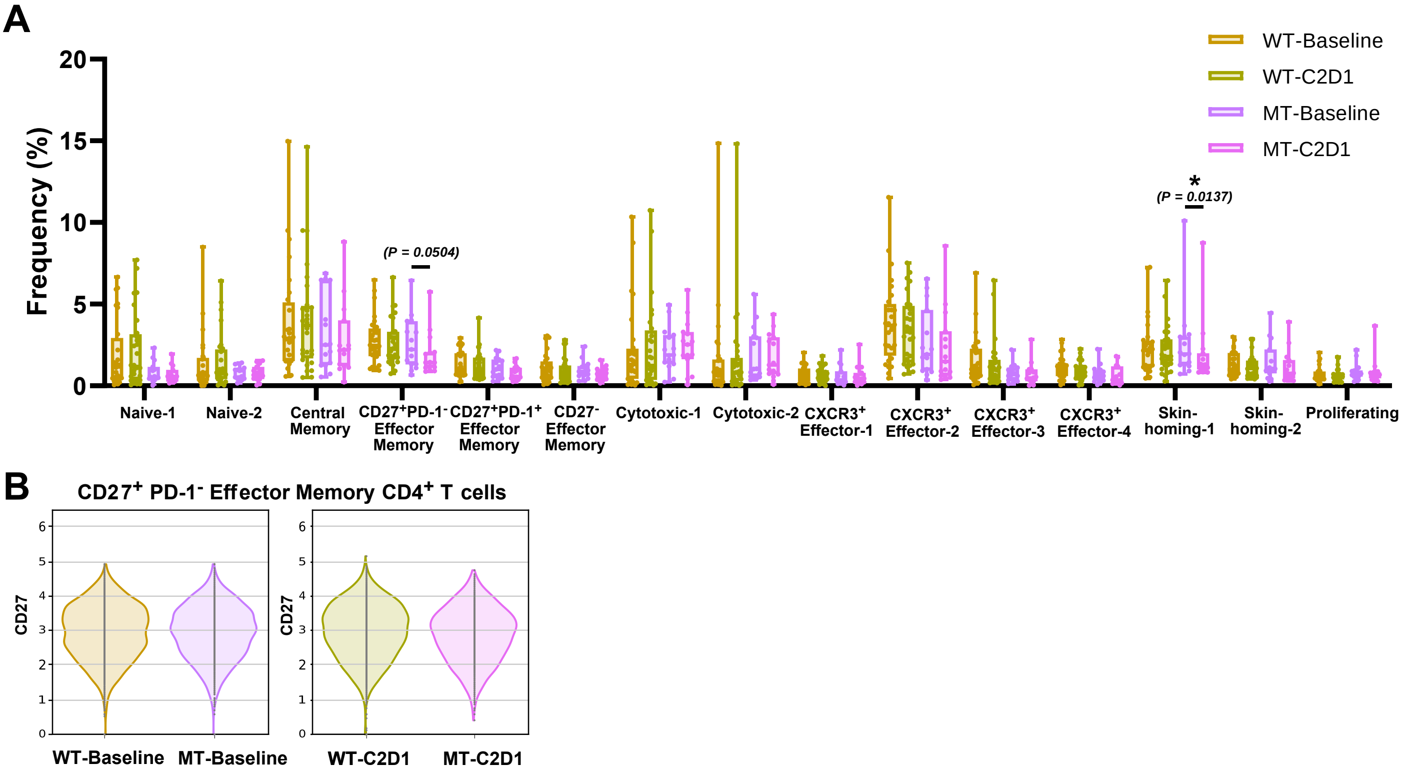

Supplementary Figure 9. Dynamic changes in CD4⁺ T cells composition before and after ICI **A.** Boxplots demonstrating the frequencies of the 15 CD4⁺ T cell subsets in WT and MT patients before and after ICI. **B.** Violin plots for CD27⁺ PD-1⁻ effector memory CD4⁺ T cells showing marker expression for CD27 at baseline and C2D1 in WT and MT patients.

Supplementary Figure 10

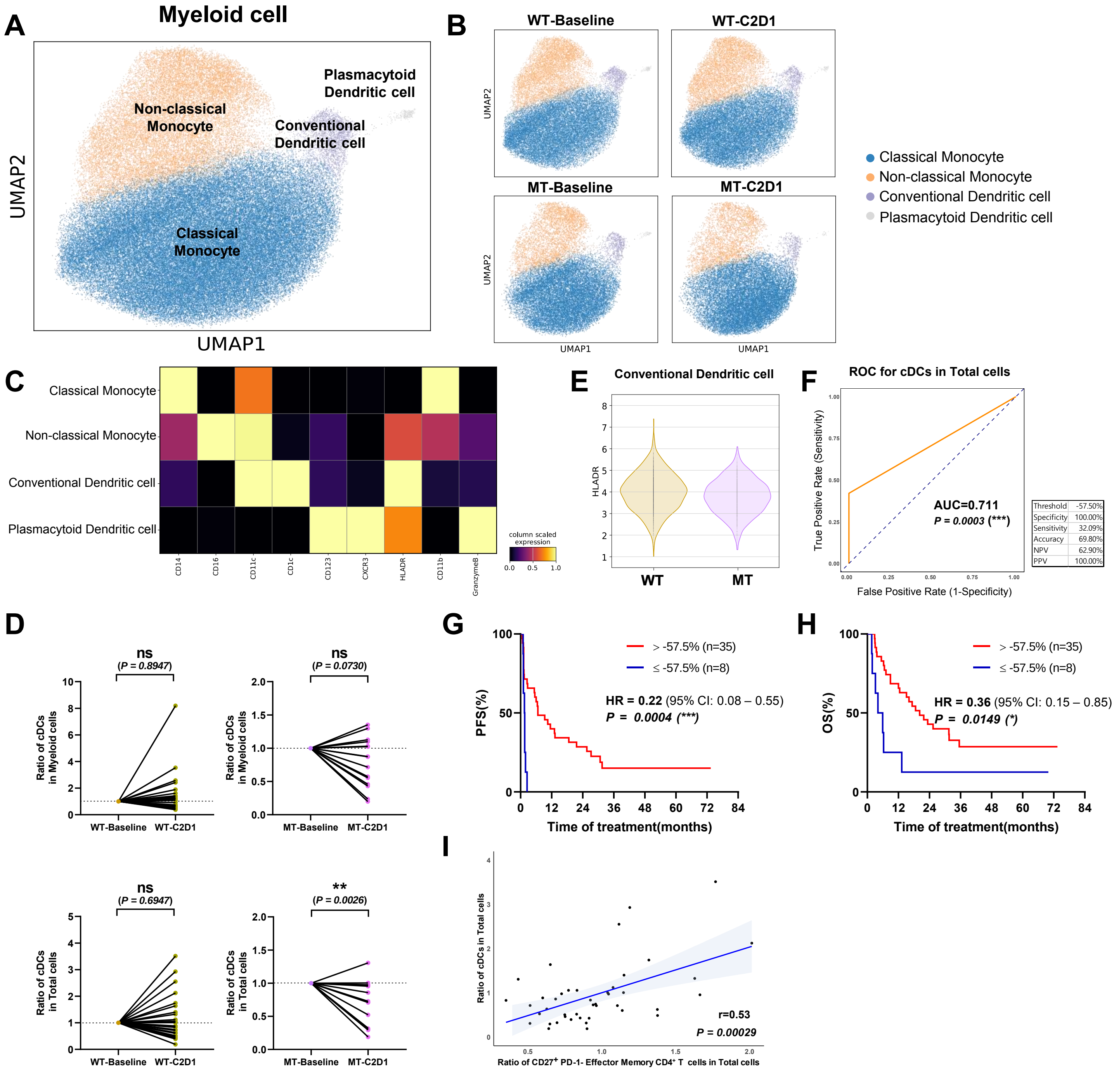

Supplementary Figure 10. Conventional DC dynamics associated with decreases in CD27 + PD-1- effector memory CD4+ T cells

A. UMAP plot identifying four myeloid cell subsets from PBMCs. **B.** UMAP plot showing the four myeloid cell subsets by mutation type and time. **C.** Heat map depicting marker expression in myeloid cell subsets. **D.** Dynamic changes in the cDCs ratio among myeloid cells and total cells in WT and MT patients before and after ICI in the discovery cohort. **E.** Violin plots of cDCs showing HLADR expression in WT and MT patients. **F.** ROC curves for predicting treatment response using the cDCs-to-total cells ratio (AUC = 0.711, n=43). **G-H.** PFS (G) and OS (H) of patients based on the reduction of the cDCs-to-total cells ratio after treatment. **I.** Pearson's rank correlation analysis comparing the cDCs-to-total cells ratio and CD27+ PD-1- effector memory CD4+ T cells-to-total live cells ratio (r = 0.53, *P < 0.001*).

Supplementary Figure 11

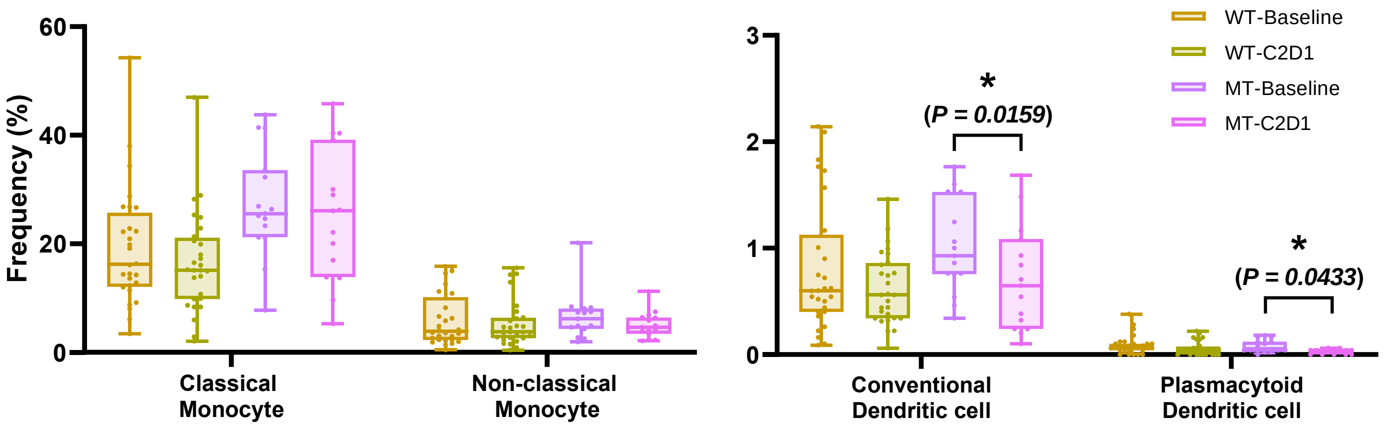

Supplementary Figure 11. Dynamic changes in the myeloid cell composition before and after ICI

A. Boxplots demonstrating the frequencies of four myeloid cell subsets among total cells in WT and MT patients before and after ICI.
